# Adult Stem Cell-Derived Intestinal Organoids as In Vitro Models of High-Fat Diet-Related Intestinal Diseases

**DOI:** 10.3390/biom16050688

**Published:** 2026-05-06

**Authors:** Yinju Liu, Lanying Chen, Zengcai Liu, Dongdong Jia, Yanlong Zhou, Jinlong Tan

**Affiliations:** 1 National Pharmaceutical Engineering Center for Solid Preparation of Chinese Herbal Medicine, Jiangxi University of Traditional Chinese Medicine, Nanchang 330006, China; 2 Jiangxi Provincial Key Laboratory of Effective Material Basis of TCM (2024SSY07102), Jiangxi University of Chinese Medicine, Nanchang 330004, China; 3 Jiangxi Province Key Laboratory of Traditional Chinese Medicine Pharmacology, Institute of Traditional Chinese Medicine Health Industry, China Academy of Chinese Medical Sciences, Nanchang 330115, China; 4 Jiangxi Health Industry Institute of Traditional Chinese Medicine, Nanchang 330115, China

**Keywords:** adult stem cell, intestinal organoid model, high-fat diet, lipid metabolism

## Abstract

The dysregulation of intestinal lipid metabolism induced by a high-fat diet (HFD) is associated with metabolic diseases; however, the validity of intestinal organoids (IOs) as substitutes for traditional research systems remains uncertain. This study aimed to determine whether fatty acid (FA)-treated IOs accurately replicate intestinal lipid metabolism observed in HFD mice. Male *C57BL/6* mice were fed either a normal chow diet (NCD) or HFD for 12 weeks, while mouse small intestinal crypt-derived IOs were treated in vitro with either an FA cocktail or a vehicle. Mice on the HFD exhibited phenotypes characteristic of metabolic syndrome, including intestinal lipid accumulation, upregulation of lipid catabolic genes, and downregulation of lipogenic genes. FA-treated IOs demonstrated enhanced budding frequency, lipid droplet accumulation, enriched lipid catabolism pathways, and suppressed lipogenesis, aligning with the in vivo findings. Omics analyses of the top 87 variable genes indicated a strong congruence between FA-treated IOs and intestinal tissues from HFD mice, with control groups clustering distinctly. The differentially expressed genes in both models were implicated in metabolic reprogramming, immune modulation, and barrier remodeling. Therefore, FA-treated IOs recapitulate key morphological and transcriptional characteristics of intestinal lipid metabolism in HFD mice, offering a valuable complementary model for investigating intestinal metabolic disorders.

## 1. Introduction

A high-fat diet induces extensive multisystem dysfunction in humans, adversely affecting metabolic, cardiovascular, and gastrointestinal systems. It contributes to obesity, type 2 diabetes mellitus, non-alcoholic fatty liver disease, and elevates the risk of cardiovascular diseases [1]. Furthermore, a high-fat diet disrupts the balance of gut microbiota, compromises the intestinal mucosal barrier, and activates chronic inflammatory pathways [2], which may result in increased intestinal permeability (commonly referred to as “leaky gut”) [3], colorectal cancer [4], and systemic metabolic inflammation [5]. In light of these potential health risks, the World Health Organization recommends regulating high-fat diets and limiting the intake of saturated and trans fats to mitigate associated adverse health effects.

Current research on the impacts of high-fat diets on human health predominantly relies on animal models, particularly the mouse model, which is the most widely utilized [6]. This dependence is based on the practical advantages of animal models, including their well-characterized genetic backgrounds, ease of experimental manipulation, and capacity to replicate complex systemic responses to dietary interventions. These traits have rendered animal models indispensable for initial mechanistic exploration over the decades [7,8,9]. However, the ethical implications of animal sacrifice raise the question: can these models be replaced? Recent policies have suggested reducing the use of experimental animals in favor of alternative in vitro models [10,11]. In exploring the molecular mechanisms of lipid and intestinal absorption, intestinal epithelial cell lines can serve as foundational model systems, especially for studies focused on single-transporter functions or basic signal transduction pathways. Nonetheless, the single-cell composition of these models lacks the complexity inherent in the multi-cellular microenvironment of intestinal tissue, which includes interactions among epithelial cells, immune cells, stromal cells, and the underlying extracellular matrix. This structural simplification may lead to biased and limited conclusions when conducting a systematic analysis of the regulatory network governing intestinal function, particularly in scenarios involving coordinated multi-cell responses to lipid overload [12]. Organoid technology, first established by Hans Clevers’ team in 2009 [13] and increasingly applied in the biomedical field since 2017 [14,15,16], has emerged as a transformative tool that addresses this gap by recapitulating tissue-specific architecture and cellular heterogeneity. This technological advancement has recently gained formal regulatory recognition. On April 10, 2025, the Food and Drug Administration (FDA) announced a significant paradigm shift: it will gradually replace animal experiments with innovative methods such as artificial intelligence and organoids in preclinical drug development and toxicology testing. This policy change not only validates the scientific rigor of organoid models but also heralds a new era in translational research for diet-related metabolic disorders.

Intestinal organoids are capable of recapitulating the fundamental structure of the intestinal epithelium in vitro, encompassing the essential crypt–villus architecture and all major cell types, including intestinal stem cells, Paneth cells, goblet cells, and enterocytes [17]. However, it remains uncertain whether intestinal organoids can accurately replicate the biological processes of the native intestine. Specifically, when modeling lipid absorption under high-fat dietary conditions, it is unclear whether the mechanisms involved align with those occurring in vivo. In this study, we compared intestinal mucosa derived from mice fed a high-fat diet with organoids cultured under high-fat diet conditions in vitro, aiming to evaluate their similarities and elucidate the relationship between organoids and their corresponding in vivo counterparts.

## 2. Materials and Methods

### 2.1. Animals and Treatment

Male *C57BL/6* mice (6–8 weeks old, *N* = 16) were housed under a 12 h light/dark cycle with free access to water. After a 1-week acclimatization period on a normal chow diet (NCD, D12450J, Research Diets), mice were randomly assigned to NCD (*N* = 8) or high-fat diet (HFD, D12492, Research Diets, 60% kcal from fat, soybean oil, *N* = 8) groups for 12 weeks. Body weight and food intake were monitored weekly. At the end point, mice were sacrificed and the tissues were collected. All the experimental procedures were performed in accordance with the Good Animal Practice Requirements of the Animal Ethics Procedures and Guidelines of the People’s Republic of China. This study was approved by the Animal Ethics Committee of the Institute of Traditional Chinese Medicine Health Industry, China Academy of Chinese Medical Sciences [approval number: 2024014], and all animal tests were conducted in strict accordance with animal welfare guidelines.

### 2.2. Tissue Sample Preparation and Histological Analysis

Liver and intestine (jejunum) tissues were collected and fixed in 4% paraformaldehyde. Next, tissues were embedded in paraffin and sectioned (5 µm thickness). Hematoxylin and eosin (H&E) staining was performed for histological evaluation. For lipid droplet visualization, frozen liver and intestine sections were stained with Oil Red O (Sigma-Aldrich, St. Louis, MA, USA) and counterstained with hematoxylin. Images were captured using a Nikon Eclipse E100 microscope (Nikon Corp., Tokyo, Japan).

### 2.3. Serum Triglyceride Test

Blood was collected retro-orbitally at week 12. Serum triglycerides (TG) were quantified using a colorimetric assay kit (BioVision, Milpitas, MA, USA) according to the manufacturer’s protocol. Absorbance was measured at 570 nm using a microplate reader (BioTek Synergy H1, BioTek Instruments, Inc., Santa Clara, CA, USA).

### 2.4. Isolation of Crypts

Intestinal crypts were isolated from the small intestine of *C57BL/6* mice. Briefly, tissues were washed in cold PBS, cut into fragments, and incubated in 2 mM EDTA for 30 min at 4 °C. Crypts were dissociated by vigorous shaking, filtered through a 70 µm strainer, and centrifuged at 290× *g* for 5 min.

### 2.5. Intestinal Organoid Culture and Intervention

Crypts were embedded in Matrigel (Corning) and cultured in Advanced DMEM/F12 medium (Gibco, Thermo Fisher Scientific Inc., Waltham, MA, USA) supplemented with 50 ng/mL EGF, 100 ng/mL Noggin, 500 ng/mL R-spondin, 10 mM HEPES, 2 mM GlutaMAX, 1× N2, and 1× B27 (all from PeproTech, Cranbury, NJ, USA). Organoids were passaged weekly. For high-fat modeling, organoids growing to day 4 were treated with FA cocktail (200 µM palmitic acid and 200 µM oleic acid in DMSO) or vehicle (DMSO) for 48 h. All organoids employed in the experiments were derived from passages no later than the fifth generation.

### 2.6. Indirect Immunofluorescence Analysis (IFA)

Organoids were fixed in 4% paraformaldehyde, permeabilized with 0.2% Triton X-100, and blocked with 10% goat serum. Primary antibodies against ATGL (1:200, Abcam, Cambridge, UK), CPT1α (1:200, Cell Signaling, Danvers, MA, USA), and FASN (1:200, Santa Cruz, Dallas, TX, USA) were incubated overnight at 4 °C, followed by Alexa Fluor-conjugated secondary antibodies (1:500, Invitrogen, Carlsbad, CA, USA). Nuclei were stained with DAPI. Images were acquired using a Leica STELLARIS 5 confocal laser scanning device (Leica, Leitz-Park in Wetzlar, Germany). Three randomly selected fluorescence images per unit area were quantified using image pro plus.

### 2.7. Nile Red Labeling for Organoids

Organoids were incubated with 1 µM Nile Red (Thermo Fisher) in PBS for 30 min at room temperature. After washing, fluorescence was visualized using a Nikon A1R confocal microscope (excitation/emission: 552/636 nm). Lipid droplets were automatically identified and labeled by the machine learning module in the high-content analysis software.

### 2.8. Transcriptomic Sequencing of Intestinal Tissues and Intestinal Organoids

Total RNA was extracted from mouse intestinal mucosa and cultured intestinal organoids (IOs) using TRIzol reagent (Invitrogen, Carlsbad, CA, USA). RNA integrity was verified using an Agilent Bioanalyzer (Agilent Technologies, Santa Clara, CA, USA). Sequencing libraries were constructed using the NEBNext Ultra II RNA Library Prep Kit (New England Biolabs, Inc., Ipswich, MA, USA). High-throughput sequencing was performed on an Illumina NovaSeq 6000 platform to generate 150 bp paired-end reads (Illumina, San Diego, CA, USA). Raw sequencing data were subjected to quality control, and clean reads were mapped to the reference genome using the STAR aligner (v2.7.11b). Differentially expressed genes (DEGs) were identified based on standardized statistical algorithms for transcriptomic comparison.

### 2.9. Hierarchical Clustering and GO Functional Enrichment Analysis

Hierarchical clustering of the top 87 variable genes (*p* < 0.05, |log2 (fold change)| > 1) was performed with Euclidean distance and complete linkage. Gene Ontology (GO) enrichment for differentially expressed genes was analyzed using DAVID (v6.8) and visualized with REVIGO (v1.8.2). Functional clusters were defined with a significance threshold of *p* < 0.05.

### 2.10. Statistical Analysis

Data are presented as mean ± SD. Differences between groups were assessed using two-tailed Student’s *t*-test or ANOVA with Tukey’s post hoc test (GraphPad Prism v9.0). *p* < 0.05 was considered statistically significant.

## 3. Results

### 3.1. Fatty Acid (FA) Induces Lipid Droplet Accumulation in IOs

**Figure 1:**
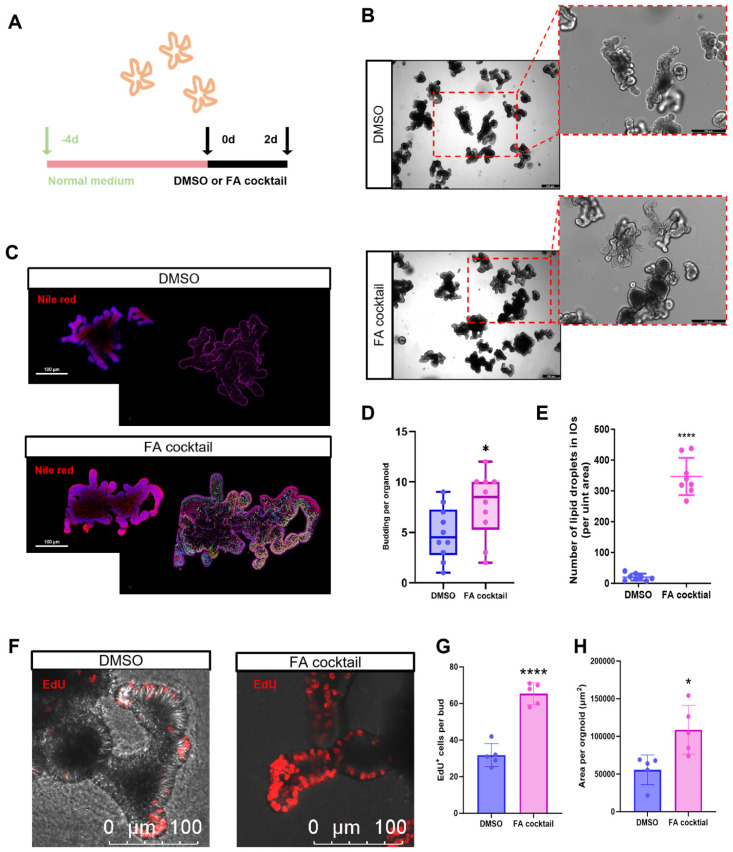
Establishment and evaluation of fatty acid (FA) cocktail-treated intestinal organoids (IOs). (**A**) Timeline of treatment IOs with DMSO and FA cocktail; (**B**) Bright-field images of IOs (The red dashed box is the magnified image, bar = 250 μm); (**C**) Nile Red staining and lipid droplet visualization analysis in IOs. The smaller image on the left shows Nile Red-labeled IOs lipid droplets, while the larger one on the right shows IOs lipid droplets labeled by the high-content analysis algorithm (bar = 100 μm); (**D**) The budding statistics of each IOs (* *p* < 0.05, *N* = 10); (**E**) Number of lipid droplets in per unit area (**** *p* < 0.0001, *N* = 8); (**F**) EdU-labeled organoids (bar = 100 μm); (**G**) The number of EdU^+^ cells per bud (**** *p* < 0.0001, *N* = 5); (**H**) Area per organoid (* *p* < 0.05, *N* = 5).

Excessive dietary lipid intake is closely associated with intestinal absorption. To evaluate whether organoids can effectively simulate the intestinal response to high-fat diets in vivo, we first constructed a fatty acid (FA) model using intestinal organoids (IOs). These IOs, derived from adult stem cells were cultured for 4 days and then treated for 2 days with either DMSO (control) or an FA cocktail (Figure 1A). Bright-field microscopy revealed that FA-treated IOs exhibited larger area (Figure 1B,H) and increased budding frequency (Figure 1D). Additionally, Nile Red staining showed a significant increase in the number of fluorescence-labeled lipid droplets in IOs in FA-treated groups (Figure 1C,E). The results of EdU labeling indicated that the proliferation level of intestinal organoids treated with FA was significantly increased (Figure 1F,G).

### 3.2. Fatty Acids Promote Intestinal Organoid Lipid Hydrolysis and Inhibit Lipid Synthesis

**Figure 2:**
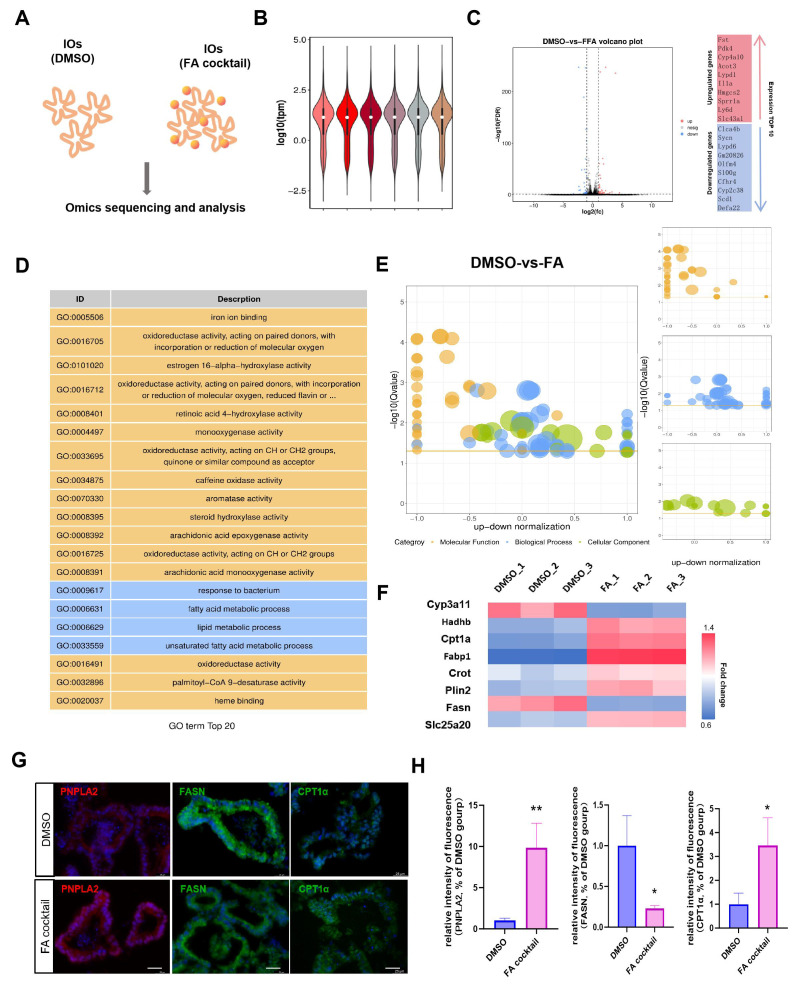
Lipid metabolism analyses intestinal organoids (IOs). (**A**) Schematic diagram of IOs collection and analysis. The orange dots indicate lipids; (**B**) Violin plots of gene expression distributions; (**C**) Volcano plot and the top 10 upregulated/downregulated of differentially expressed genes between DMSO and FA cocktail-treated IOs. Red dots represent significantly upregulated genes, blue dots represent significantly downregulated genes, and black dots represent genes with no significant difference; (**D**) Top 20 enriched Gene Ontology (GO) terms of differentially expressed genes, categorized by molecular function (yellow) and biological process (blue); (**E**) Bubble plot of GO enrichment analysis for differentially expressed genes in DMSO vs FA cocktail group, covering molecular function, biological process and cellular component. (The bubble size represents the number of enriched genes, and the color indicates the GO category: yellow bubbles indicate molecular function, blue bubbles indicate biological process, and green bubbles indicate cellular component); (**F**) Heat map of representative lipid metabolism-related marker genes in DMSO and FA cocktail-treated IOs. The color scale indicates the relative expression level (red: high expression, blue: low expression); (**G**) Immunofluorescence analysis of representative lipid metabolism proteins (bar = 25 μm); (**H**) Relative fluorescence intensity of PNPLA2, FASN and CPT1α labeling per unit area (* *p* < 0.05, ** *p* < 0.01, *N* = 3).

Transcriptomic profiling of DMSO/FA-treated intestinal organoids (IOs, Figure 2A) produced high-quality datasets. Sample homogeneity and data quality were assessed using violin plots displaying gene expression distributions (Figure 2B). To identify transcriptomic alterations induced by fatty acid challenge, a volcano plot was constructed to display the expression landscape of differentially expressed genes (DEGs) between the DMSO control and FA cocktail-treated groups, in which red dots represent significantly upregulated genes and blue dots represent significantly downregulated genes upon FA treatment, with the top 10 upregulated and downregulated genes annotated alongside the plot (Figure 2C). To further explore the biological functions of these differentially expressed genes (DEGs), Gene Ontology (GO) enrichment analysis was conducted, identifying the top 20 significantly enriched functional terms categorized into molecular function (yellow) and biological process (blue), with pathways related to lipid metabolism showing prominent enrichment (Figure 2D). To gain a comprehensive overview of functional enrichment, a GO bubble plot analysis was subsequently performed, encompassing molecular function, biological process, and cellular component. In this plot, the size of the bubbles corresponds to the number of enriched genes, while color denotes the GO category: yellow for molecular function, blue for biological process, and green for cellular component (Figure 2E). A heatmap was then generated to illustrate the expression patterns of representative lipid metabolism-related marker genes, revealing distinct expression profiles between the DMSO and FA treatment groups. Specifically, genes involved in lipolysis and mitochondrial β-oxidation, such as *Cyp3a11* and *Cpt1a*, were significantly upregulated, while key lipogenic genes, including *Fabp1* and *Fasn*, were markedly downregulated in response to FA cocktail stimulation (Figure 2F). To validate the transcriptomic results at the protein level, immunofluorescence staining was performed, confirming a significant increase in the expression of PNPLA2, a key marker of FA-induced lipolysis, and CPT1α, the rate-limiting enzyme of mitochondrial β-oxidation, alongside a notable reduction in FASN, a critical lipogenic marker. These gene-level changes were fully consistent with the transcriptomic data (Figure 2G,H). These characteristics of metabolic reprogramming align with established pathophysiological mechanisms associated with high-fat diets, underscoring the fidelity of IOs in modeling diet-induced intestinal pathology.

### 3.3. Lipid Accumulation in the Intestinal Villi of Obese Mice

**Figure 3:**
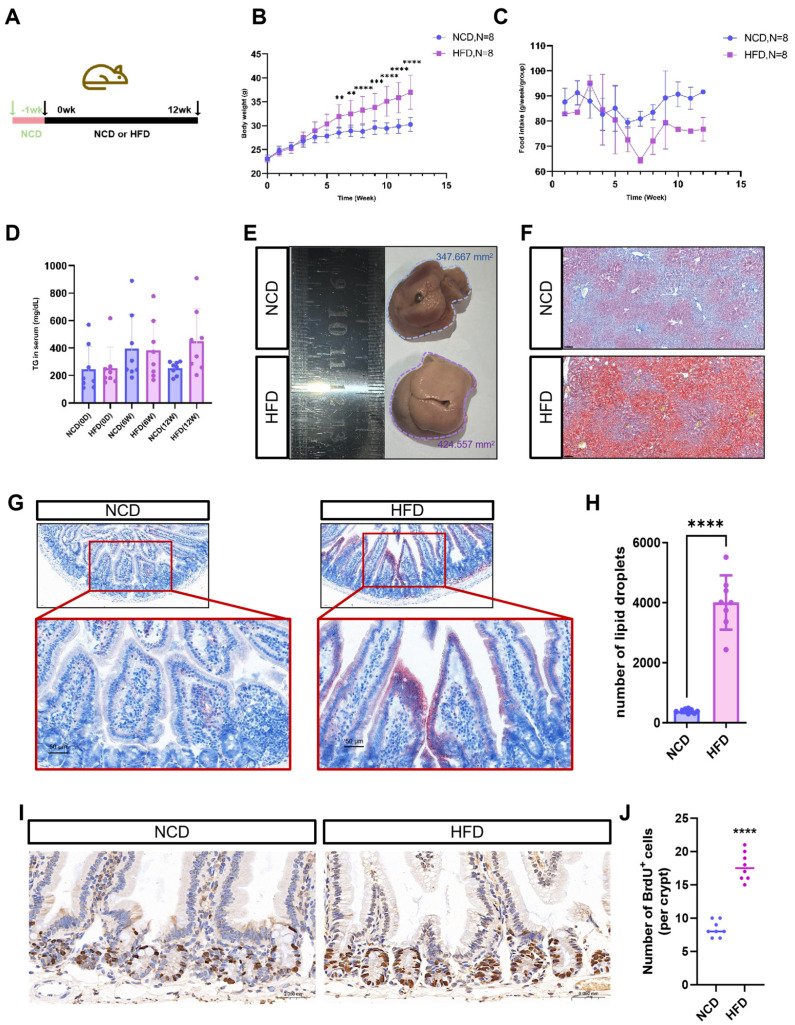
Establishment and evaluation of high-fat diet animal model. (**A**) Timeline of feeding normal chow diet (NCD) and high-fat diet (HFD) to mice; (**B**) The curve of weight change in mice (the blue circles indicate the NCD group, and the purple squares indicate the HFD group, ** *p* < 0.01, *** *p* < 0.001, **** *p* < 0.0001, *N* = 8); (**C**) The curve of weekly feed intake per group (the blue circles indicate the NCD group, and the purple squares indicate the HFD group, *N* = 8). There was no significant difference between the two groups at different times; (**D**) The variation in the change in triglyceride (TG) in serum (*N* = 8); (**E**) Bright-field images of isolated liver (The blue-dotted circle indicates the maximum area of the liver in the NCD group as viewed from above, and the purple-dotted circle indicates the maximum area of the liver in the HFD group as viewed from above.); (**F**) Oil Red O staining of the mouse liver tissue (bar = 100 μm); (**G**) Oil Red O staining of the mouse intestinal tissue (The red-dashed box is the magnified image, bar = 50 μm); (**H**) Graph of lipid droplet count in mouse intestinal tissue section (**** *p* < 0.0001, *N* = 8); (**I**) Immunohistochemistry of BrdU-labeled intestinal tissue (bar = 0.050 mm); (**J**) The number of BrdU^+^ cells in one single crypt (*N* = 8. **** *p* < 0.0001, *N* = 8).

To evaluate the efficacy of organoids in simulating the intestinal response to high-fat diets in vivo, we constructed a high-fat animal model. Sixteen *C57BL/6* mice, aged 6–8 weeks, were acclimated for one week under standard conditions, which included 12 h light/dark cycles, ad libitum access to water, and a normal chow diet (NCD). During the modeling phase, the mice were randomized into NCD and high-fat diet (HFD) groups (Figure 3A). We monitored body weight, food intake, and triglyceride (TG) levels. Notably, HFD mice exhibited significantly higher body weights than their NCD counterparts starting from week 6 (Figure 3B), despite a reduction in appetite during later stages (Figure 3C). By week 12, HFD mice displayed elevated serum TG levels (Figure 3D). Observations made post-sacrifice revealed that the livers of NCD mice appeared reddish-brown, while those of HFD mice were pale yellow and enlarged (Figure 3E). Histological analysis of liver sections further confirmed the presence of severe steatosis in HFD mice, as indicated by Oil Red O staining, which demonstrated diffuse lipid droplet accumulation, narrowed central veins, hemosiderin deposition, and indistinct hepatic sinusoids (Figure 3F). Collectively, these findings indicate the successful establishment of a high-fat-induced fatty liver model. Comparative Oil Red O staining of intestinal tissues revealed greater lipid droplet accumulation in the epithelia and submucosa of HFD mice compared to NCD controls (Figure 3G). The number of lipid droplets within the intestinal villi was markedly elevated in HFD mice (Figure 3H). Furthermore, the quantity of BrdU-labeled cells in the intestines of HFD mice was significantly higher than that in NCD mice (Figure 3I,J), suggesting an enhanced intestinal proliferation capacity in mice.

### 3.4. Elevated Lipid Hydrolysis and Reduced Lipid Synthesis in Intestinal Villi of Obese Mice

**Figure 4:**
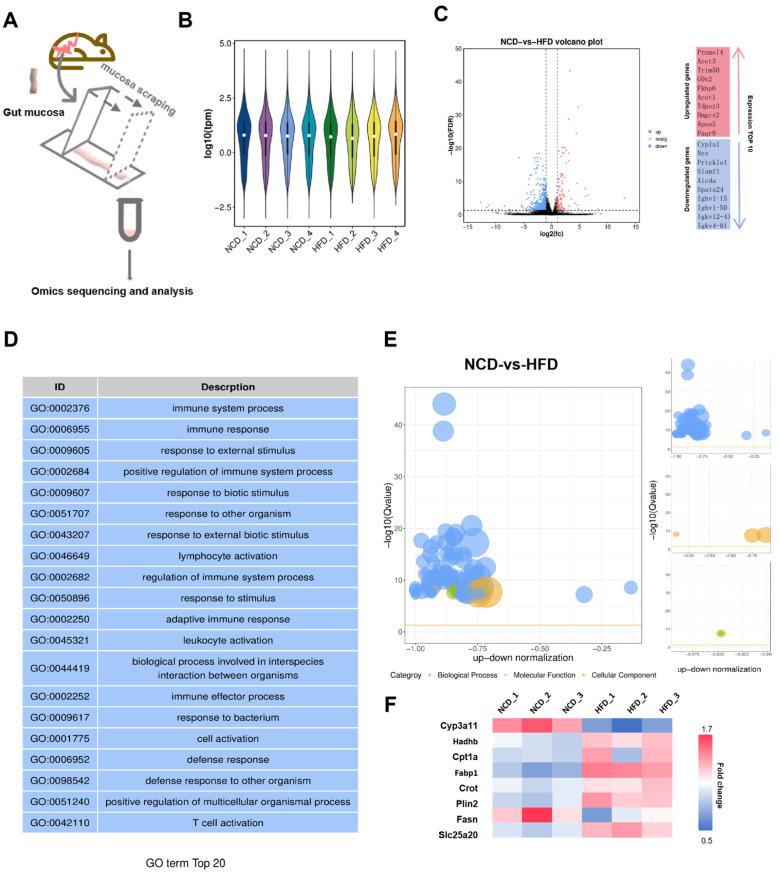
Lipid metabolism analyses in intestinal tissue. (**A**) Schematic diagram of mucosa collection and analysis; (**B**) Violin plot of gene expression levels; (**C**) Volcano plot and the top 10 upregulated/downregulated differentially expressed genes (DEGs) between the NCD and HFD groups. Red dots represent significantly upregulated genes in HFD, blue dots represent significantly downregulated genes in HFD, and gray dots represent genes with no significant difference; (**D**) Top 20 enriched Gene Ontology (GO) terms of differentially expressed genes, primarily categorized into biological process (blue background), highlighting immune system-related functional enrichments; (**E**) Bubble plot of GO enrichment analysis for differentially expressed genes in the NCD vs HFD group. (The bubble size represents the number of enriched genes, and the color indicates the GO category: yellow bubbles indicate molecular function, blue bubbles indicate biological process, and green bubbles indicate cellular component); (**F**) Heat map of representative lipid metabolism-related marker genes in NCD and HFD groups. The color scale represents the relative expression level (red: high expression, blue: low expression).

To delineate the intestinal mucosal transcriptomic landscape in response to dietary lipid overload, RNA sequencing (RNA-seq) was conducted on intestinal mucosa harvested from mice maintained on either a normal chow diet (NCD) or a high-fat diet (HFD) (Figure 4A). Mucosal samples were isolated through targeted mechanical scraping, followed by strand-specific library construction and paired-end high-throughput sequencing. The complete transcriptomic raw datasets have been deposited as Supplementary Table S1. Sample homogeneity and data quality were validated through violin plots showing transcript expression distributions (Figure 4B). A volcano plot was generated to identify the global transcriptomic changes between NCD and HFD groups, revealing a substantial number of differentially expressed genes (DEGs) with a threshold of |log2 (fold change)| > 1 and adjusted *p*-value < 0.05: red dots represented significantly upregulated genes in HFD, while blue dots indicated significantly downregulated genes, with black dots showing no significant expression difference, and the top 10 upregulated and downregulated genes annotated alongside the plot (Figure 4C). Subsequent Gene Ontology (GO) enrichment analysis of DEGs identified the top 20 enriched biological process terms, which were predominantly associated with immune system function, including immune system process, immune response, response to external/biotic stimulus, lymphocyte activation, adaptive immune response, and T cell activation (Figure 4D). Further GO bubble plot analysis was performed to comprehensively characterize the functional enrichment of DEGs, covering biological process, molecular function, and cellular component (Figure 4E). In accordance with the classification in Figure 2E, yellow bubbles represent molecular function, and blue bubbles represent biological process, without indicating gene upregulation or downregulation. The bubble size represents the number of enriched genes, and the color intensity reflects the statistical significance of enrichment. This visualization further confirmed the major functional clusters perturbed by HFD exposure. Consistent with our prior observations in fatty acid (FA)-challenged intestinal organoids (IOs), the focus was placed on lipid metabolism-related genes to dissect the dietary modulation of intestinal lipid homeostasis. A heatmap of representative lipid metabolism-related marker genes illustrated distinct expression patterns between NCD and HFD groups (Figure 4F). Genes associated with lipid synthesis pathways were significantly repressed in HFD-exposed intestinal crypts, with *Cyp3a11* and *Fasn* exhibiting marked downregulation in HFD samples. This attenuation of endogenous lipogenesis likely represents an adaptive response aimed at reducing intestinal lipid production under conditions of excess dietary lipid supply. In contrast, lipid catabolic pathways, which coordinate fatty acid breakdown and oxidative energy generation, were robustly upregulated in HFD-fed mice. Key regulatory effectors included *Hadhb*, *Cpt1a*, and *Crot*. Collectively, these genes orchestrate lipid hydrolysis, mitochondrial trafficking, and oxidative degradation; their elevated expression in HFD mucosa indicates a compensatory enhancement of lipid catabolism, a mechanism likely evolved to mitigate lipid accumulation in the intestinal epithelium. Differential expression was also observed for accessory lipid-handling genes, such as *Fabp1*, which encodes an intracellular fatty acid chaperone that mediates cytoplasmic lipid transport, and *Plin2* [18], a lipid droplet-associated structural protein that regulates lipid droplet dynamics [19]. This further confirms the coordinated rewiring of intestinal lipid metabolic networks in response to a HFD (Figure 4F).

### 3.5. Consistent Omics Profiles of FA-Treated Intestinal Organoids and HFD-Fed Mouse Intestinal Epithelium

**Figure 5:**
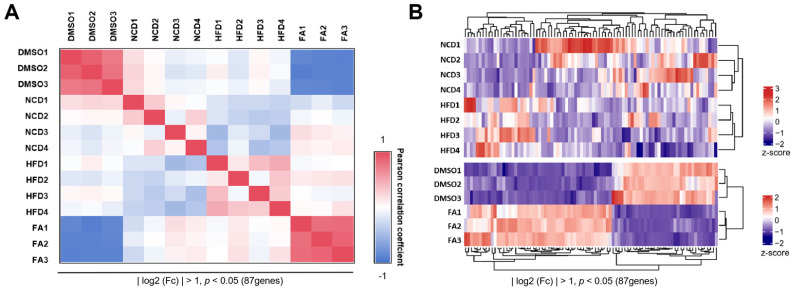
Combined Transcriptomic Analysis of Intestinal Tissues and Intestinal Organoids (IOs). (**A**) Pearson correlation analysis. The top 87 variable genes were based on statistical significance (*p* < 0.05) and |log2 (fold change)| > 1; (**B**) Heatmap represents hierarchical clustering of mice and IOs samples based on the expression of dysregulated proteins in healthy and HFD intestinal mucosa (top) and dysregulated genes in DMSO and FA-treated IOs (bottom). The top 87 variable genes were based on statistical significance (*p* < 0.05) and |log2 (fold change)| > 1.

To elevate the consistency between fatty acid (FA)-treated intestinal organoids (IOs) and HFD-fed mouse models, we z-score-normalized the gene expression data and calculated Pearson correlation coefficients for the 87 most variable genes (*p* < 0.05, |log2 (fold change)| > 1 relative to the control; listed in Supplementary Table S2). This subset effectively captures the core molecular responses to lipid overload. The Pearson correlation heatmap (Figure 5A) illustrated distinct clustering that corresponded to the lipid exposure groups: DMSO-treated IOs (in vitro control) clustered with normal chow diet (NCD)-fed mouse intestinal tissues (in vivo control), exhibiting strong positive correlations (Pearson r ≈ 1). In contrast, FA-treated IOs (in vitro lipid challenge) correlated with HFD-fed tissues (in vivo lipid challenge) and showed negative correlations with controls. This pattern confirms that the IOs model recapitulates key aspects of in vivo intestinal transcriptomic responses to high-lipid conditions. Hierarchical clustering of the same 87 genes (Figure 5B) further validated this concordance, revealing a binary stratification: control samples (DMSO/IOs, NCD tissues) were grouped in one branch, while high-lipid samples (FA/IOs, HFD tissues) formed another. Within each branch, in vitro and in vivo samples of the same lipid condition clustered closely, with large Euclidean distances observed between opposing groups (e.g., DMSO/IOs vs. FA/IOs). Collectively, these analyses demonstrate a strong transcriptomic congruence between FA-treated IOs and HFD mouse models.

## 4. Discussion

Exposure to a high-fat diet (HFD) induces systemic and intestinal lipid metabolic dysregulation in mice, as evidenced by the emergence of canonical metabolic syndrome phenotype, including increased body mass, hepatic steatosis, and elevated serum triglycerides. At the intestinal level, HFD prompts adaptive remodeling to manage lipid overload, characterized by villus atrophy and submucosal lipid deposition. These morphological changes are accompanied by functional shifts in lipid metabolism, chronic high-fat exposure, which enhances intestinal stem cell activity, accelerates villus development and differentiation, and increases the risk of colorectal cancer [20,21]. Collectively, these phenotypic and functional alterations establish HFD mice as a well-validated in vivo model for studying lipid-induced intestinal metabolic perturbation.

Fatty acid (FA)-treated intestinal organoids (IOs) replicate lipid-responsive phenotypes that mirror the intestinal adaptations observed in HFD mice. Phenotypically, FA-exposed IOs exhibit hypertrophic growth, significant lipid droplet accumulation, and increased branching compared to control organoids—morphological hallmarks that align with the enhanced intestinal stemness and differentiation triggered by HFD in vivo. Beyond gross morphology, FA-treated IOs demonstrate molecular reprogramming consistent with lipid stress: omics analyses confirm that these organoids display aberrant lipid metabolism signatures analogous to HFD-exposed intestinal crypts, validating that exogenous FA exposure recapitulates the core lipid metabolic perturbations induced by dietary lipid excess in animal models.

The concordance between HFD mice and FA-treated IOs extends beyond isolated phenotypic traits to encompass molecular and functional alignment, thereby confirming the physiological relevance of the organoid model for lipid metabolism research. Omics integration revealed a robust transcriptomic overlap between FA-treated IOs and HFD intestinal tissue, with control groups clustering distinctly. GO-based functional annotation of differentially expressed genes (DEGs) further corroborated this congruence: lipid catabolism pathways, such as *ATGL* and *CPT1α* gene upregulation, were enriched in FA-treated organoids, corresponding to quadrants Q1–Q2. This pattern is indicative of compensatory energy mobilization under caloric excess. In contrast, the signatures in quadrants Q3–Q4 were characterized by suppressed glucose transport-related gene expression, reduced immune modulation (e.g., *IL-8* gene downregulation), and basement membrane remodeling-related gene dysregulation, all of which align with results reported in previous research [22,23]. These molecular and functional shifts mirror well-characterized HFD-induced pathophysiology, such as gut barrier dysfunction and meta-inflammation, demonstrating that FA-treated IOs capture not only morphological but also mechanistic aspects of lipid adaptation in vivo. Collectively, this study employs a multi-dimensional validation strategy: morphological changes and proliferation markers (EdU, BrdU) confirm the model’s fidelity regarding growth dynamics and structural adaptation. In contrast, transcriptomic data—encompassing lipid metabolism-related genes and associated GO terms—offer insights at the metabolic and functional levels. These two layers of evidence are complementary, collectively supporting the conclusion that organoids effectively recapitulate key aspects of in vivo intestinal responses to high-lipid conditions.

Organoids have long been recognized for their capacity to recapitulate the structural architecture of adult organs. For instance, intestinal organoids self-assemble into structured epithelia containing enterocytes, endocrine cells, Paneth cells, and *Lgr5*^+^ stem cells [17]. Similarly, liver organoids reconstruct niches for hepatic stellate cells, cholangiocytes, and progenitor cells [9]. Vascular organoids, on the other hand, form perfusable networks with pericytes and smooth muscle cells enveloping endothelia [24]. Critically, this structural mimicry extends to pathological relevance: vascular organoids exposed to high glucose and inflammatory factors replicate the thickening of the diabetic basement membrane [25]. iPSC-derived liver organoids develop functional bile canalicular systems that are compromised by cholestatic drugs, such as troglitazone, and they also recapitulate phenotypes associated with non-alcoholic steatohepatitis (NASH) following FA incubation [26]. Furthermore, vascular organoids recapitulate key vascular pathological features, such as endothelial barrier dysfunction at the molecular, cellular, and tissue levels [27]. Our findings contribute to this body of evidence by demonstrating that IOs not only replicate intestinal structural composition but also closely mirror key lipid-induced pathophysiological responses, further validating organoids as pathologically relevant complementary models to in vivo tissues.

While organoids hold significant promise for mimicking organ functionality and reducing reliance on animal experimentation, comprehensive evidence validating their equivalence to in vivo systems—beyond structural similarity to a “bionic effect”—has remained limited. Intestinal organoids have been employed to establish models of inflammatory bowel disease (IBD) [28], alveolar organoids to recapitulate the pathological processes of COVID-19 infection [29,30], and cancer organoids to facilitate personalized precision therapy [31,32]. However, direct evidence verifying the functional and phenotypic concordance between organoid models and target organs remains scarce. Our study addresses this gap by integrating phenotypic and transcriptomic analyses across murine in vivo and organoid in vitro systems, demonstrating that FA-treated IOs phenocopy key pathophysiological features of dietary lipid overload observed in HFD mice across morphological and transcriptomic dimensions. The translational relevance of the IOs system for studying intestinal lipid metabolism is supported by this cross-model concordance, with its utility as a surrogate for in vivo tissue responses to validated high-fat environments. This multi-layered concordance positions IOs as scalable, reductionist platforms for dissecting epithelial-intrinsic lipid responses to dietary challenge. While lacking the full complexity of in vivo systems—including immune and stromal components—IOs offer clear utility as complementary tools for accelerating mechanistic research and therapeutic screening for metabolic disorders.

Notably, our transcriptomic results revealed substantial differences in the top 20 enriched GO terms between intestinal tissues and intestinal organoids, highlighting inherent distinctions in global gene expression profiles between the in vivo and in vitro systems. It is important to note that the primary intestinal crypts utilized for transcriptomic analysis inherently contain resident immune cells, including macrophages, lymphocytes, and dendritic cells, which are integral components of the intestinal mucosal immune microenvironment. These resident immune cells can actively participate in the transcriptional response to dietary lipid overload, thus biasing Gene Ontology (GO) and Kyoto Encyclopedia of Genes and Genomes (KEGG) enrichment results towards immune-related pathways (e.g., immune response, lymphocyte activation) that are detectable in the in vivo crypt transcriptome. In contrast, the intestinal organoid model, derived from intestinal crypt stem cells and primarily composed of epithelial cells (enterocytes, Paneth cells, endocrine cells), lacks this complex immune microenvironment (see Figure 2D and Figure 4D). This compositional difference—specifically, the presence of immune cells in vivo crypts versus their absence in vitro organoids—represents a key distinction between the two systems. Notably, the immune-dominated GO enrichment in tissue samples also indicates that HFD exerts a significant impact on the mucosal immune compartment, a dimension that cannot be captured by the current epithelium-only organoid model. Therefore, a direct and detailed comparison of the complete crypt transcriptome with the organoid transcriptome may be confounded by this inherent cellular heterogeneity, as immune cell-derived transcriptional signals in the crypt samples may not be reflected in the organoid samples. To address this potential confounding factor and ensure a meaningful, targeted comparison of core intestinal epithelial responses between the two systems, our analysis intentionally focused on conserved lipid metabolism markers and epithelial-centric pathways (e.g., lipid catabolism, lipogenesis, intestinal barrier function), which are primarily driven by epithelial cells and independent of immune cell contributions. This targeted approach enabled us to eliminate the interference of immune cell-derived transcriptional signals, thereby accurately evaluating the concordance of lipid metabolic responses between fatty acid (FA)-treated intestinal organoids (IOs) and high-fat diet (HFD)-induced intestinal tissues. However, it should be noted that the current in vivo analysis relied primarily on transcriptomic profiling; direct functional measurements of immune cell activity or metabolic flux were not performed, which represents a limitation of this study. Future investigations incorporating such functional assays will be necessary to validate the mechanistic implications of the observed transcriptional changes.

Another important consideration pertains to the temporal divergence between the two models: the in vivo HFD model represents a chronic metabolic challenge sustained over 12 weeks, whereas the in vitro IOs model employs an acute 48 h fatty acid treatment. Although both paradigms elicit comparable phenotypic outcomes, such as intracellular lipid accumulation and enhanced lipid catabolic signaling, the underlying molecular programs may diverge substantially. Chronic HFD exposure in vivo is known to drive progressive adaptations including low-grade inflammation, cellular senescence, and long-term epithelial remodeling, whereas acute FA treatment in vitro primarily triggers an immediate stress response and early metabolic adaptation. Despite these temporal and mechanistic differences, the core epithelial-intrinsic lipid metabolic programs remain highly conserved between the two systems. This supports the utility of acute FA exposure for modeling early epithelial responses to lipid overload, although prolonged in vitro models may better capture chronic adaptive processes. Collectively, these findings provide substantive evidence supporting organoids as viable alternatives to animal models in lipid metabolism research. Nevertheless, the robust congruence between FA-treated IOs and HFD mice supports the use of organoids as complementary (and in select contexts, substitutive) tools for preclinical research, balancing physiological relevance with the ethical and practical benefits of reducing animal experimentation.

## 5. Conclusions

This study demonstrates that FA-treated adult stem cell-derived IOs capture key morphological, transcriptomic features of intestinal lipid metabolism dysregulation observed in HFD-fed *C57BL/6* mice. HFD mice exhibited metabolic syndrome phenotypes, intestinal lipid accumulation, upregulated lipid catabolic genes (e.g., *Hadhb*, *Cpt1a*) and downregulated lipogenic genes (e.g., *Fasn*), while FA-treated IOs showed increased diameter, budding frequency, lipid droplet accumulation, enriched lipid catabolism pathways and suppressed lipogenesis—consistent with in vivo findings. Multi-omics analyses of top 87 variable genes confirmed strong congruence between FA-treated IOs and HFD mouse intestinal tissues, with differentially expressed genes involved in metabolic reprogramming, immune modulation and barrier remodeling. In conclusion, we demonstrate that FA-induced IOs model provides a useful complementary platform for investigating epithelial aspects of HFD-related intestinal metabolic disorders and accelerating preclinical drug screening, thereby advancing translational research in metabolic health.

## Data Availability

The original contributions presented in this study are included in the article. The raw RNA-seq datasets generated and analyzed during the current study have been deposited as Supplementary Table S1. Further inquiries can be directed to the corresponding author. During the preparation of this manuscript, the authors used Deepseek V4 for language polishing, grammar checking, and improving readability. These tools were used solely for linguistic refinement and not for generating, analyzing, or interpreting scientific content. The authors take full responsibility for the intellectual integrity and scientific accuracy of the work.

## Supplementary Materials

The following supporting information can be downloaded at: https://www.mdpi.com/article/10.3390/biom16050688/s1, Table S1: Complete raw data; Table S2: Screened genes data.
